# The model of circulating immune complexes and interleukin-6 improves the prediction of disease activity in systemic lupus erythematosus

**DOI:** 10.1038/s41598-018-20947-4

**Published:** 2018-02-08

**Authors:** Chokchai Thanadetsuntorn, Pintip Ngamjanyaporn, Chavachol Setthaudom, Kenneth Hodge, Nisara Saengpiya, Prapaporn Pisitkun

**Affiliations:** 1 Division of Allergy Immunology and Rheumatology, Department of Medicine, Faculty of Medicine Ramathibodi Hospital, Mahidol University, Rama VI Road, Bangkok, 10400 Thailand; 2 Immunology Laboratory, Department of Pathology, Faculty of Medicine Ramathibodi Hospital, Mahidol University, Rama VI Road, Bangkok, 10400 Thailand; 3 Center of Excellence in Systems Biology, Faculty of Medicine, Chulalongkorn University, Bangkok, 10330 Thailand

## Abstract

Systemic Lupus Erythematosus (SLE) is an autoimmune disease resulting in autoantibody production, immune complex deposition, and complement activation. The standard biomarkers such as anti-dsDNA and complements (C3 and C4) do not always correlate with active clinical SLE. The heterogeneity of SLE patients may require additional biomarkers to designate disease activity. Ninety SLE patients participated in this study. Evaluation of disease activity was achieved with the Systemic Lupus Erythematosus Disease Activity Index 2000 (SLEDAI-2K) and modified SLEDAI-2K. The measured serum biomarkers were anti-dsDNA, C3, C4, ESR, interleukin-6 (IL-6), and circulating immune complexes (CIC). IL-6, ESR and CIC significantly increased in active clinical SLE. Complement, anti-dsDNA, ESR and CIC correlated with SLEDAI-2K while only anti-dsDNA, CIC, ESR and IL-6 correlated with modified SLEDAI-2K. A combination of biomarkers gave a higher odds ratio (OR) than any single biomarker. A combination of IL-6 or CIC exhibited the highest OR (OR = 7.27, 95%CI (1.99–26.63), p = 0.003) while either complement or anti-dsDNA showed a moderate odds ratio (OR = 3.14, 95%CI (1.16–8.48), p = 0.024) of predicting clinical active SLE. The combination of CIC and IL-6 strongly predicts active clinical SLE. CIC and IL-6 can be used in addition to standard biomarkers to determine SLE activity.

## Introduction

Systemic Lupus Erythematosus (SLE) is an autoimmune disease affecting all organ systems leading to inflammation and tissue damage^1^. The classical immunological abnormalities found in SLE are autoantibody production, immune complex deposition and complement activation^2^. The pathogenesis of SLE is complex and involves several genetic abnormalities which result in heterogeneity in disease manifestations^3^. The outcomes of SLE progression in patients can be devastating, with cumulative morbidity over time from disease activity or infectious complication^4^. Most of the clinical trials conducted with SLE patients fail to show potency of the treatment to control disease activity, although some treatments showed efficacy in some case reports^5^. One confounding problem may be the lack of sufficiently sensitive tools to monitor SLE disease activity. Accurate assessment of SLE disease activity is necessary to help physicians differentiate active disease from chronic non-inflammatory injury and provide the patient with appropriate treatment.

Beyond genetics and environmental factors, cytokine dysregulation is ubiquitous, and their proteins and gene expression profiles may serve as markers of disease activity and severity. From previous studies, the key cytokine involved in SLE pathogenesis is interferon alpha (IFN- α) which leads to upregulation of several inflammatory proteins^6^. Additionally, IL-6, TNF-α, IFN-γ, and BLyS, as well as T-cell-derived cytokines like IL-17, IL-21, and IL-2, are dysregulated in SLE^3,7^.

IL-6 can promote activation and differentiation of cells central to the development of systemic autoimmunity and the associated pathologic inflammatory responses^8^. There is evidence that serum levels of IL-6 are elevated in human SLE and have correlated with disease activity or anti-dsDNA levels in some studies^9–11^. However, IL-6 production in LPS stimulated whole blood culture was lower in SLE patients compared to controls in another study^12^. IL-6 closely linked with specific disease manifestations of SLE patients. Urinary IL-6 correlates with titers of anti-dsDNA antibodies and decreases following treatment in patients with lupus nephritis. Also, the expression of IL-6 increased in glomerular and tubular tissue in lupus nephritis kidneys^13^. On the other hand, another study showed higher levels of IL-6 in SLE with hematological manifestation, but did not correlate with other individual organ and systemic disease activity^14^.

Elevated serum levels of circulating immune complexes (CIC) have long been described in lupus, which leads to organ inflammation and damage by immune complex deposition. Immune complexes are composed of circulating DNA and antibodies to DNA^15^. Few studies show the detection of CIC is specific for SLE, and correlates with disease activity and deposits in the kidney of lupus nephritis patients^16,17^. Several methods can be used to detect CIC, but no single procedure appears to detect all types of CICs^18^. Those procedures which detect CIC’s containing fragments of complement (e.g. C1q and C3d) seem to match with clinically relevant events^19^.

There exists conflicting data on the correlation between SLE disease activity with serum IL-6 and CIC, and those studies have been performed in small groups of patients, while others are retrospective studies^9–11,17,18^. This study aimed to investigate whether serum levels of IL-6 and CIC correlated with SLE disease activity achieved by the Systemic Lupus Erythematosus Disease Activity Index (SLEDAI-2K) and modified SLEDAI-2K. If so, the analysis of biomarker models would be compared to reveal the model that provides the best prediction for SLE disease activity.

## Results

### Patient demographics and clinical characteristics

Ninety SLE patients were enrolled in the study. Active disease was defined if the clinical SLEDAI scores were greater than one. Of the 90 total patients, 27 cases (30%) had active clinical SLE whereas 63 cases (70%) had inactive clinical SLE. The majority of patients were female (93.3%), and median disease duration was 83.5 months. No statistically significant difference was observed regarding gender, disease duration and underlying diseases between the active and inactive group, except the age of active SLE group was significantly lower than the inactive SLE group (32.67 ± 13.34 years versus 43.35 ± 14.24 years, *p* = 0.001). The active SLE group had a statistically significant higher erythrocyte sedimentation rate (ESR) and urine protein to creatinine ratio, but lower absolute lymphocyte counts compared with patients in the inactive SLE group. Also, the active SLE group had a statistically significant higher number of patients receiving prednisolone, mycophenolate mofetil and tacrolimus than the inactive group (Table 1). The prevalence of activity of all 24 descriptors of SLEDAI-2K for the studied cohort is shown (Supplementary Table S1).

**Table 1:** Patient demographics and clinical characteristics.

Clinical parameters	Total (n = 90)	Active SLE (n = 27)	Inactive SLE (n = 63)	*p* value
Age, year (mean ± SD)**	40.14 ± 14.75	32.67 ± 13.34	43.35 ± 14.24	0.001
Gender				
Male, n (%)	6 (6.7)	4 (14.8)	2 (3.2)	0.064
Female, n (%)	84 (93.3)	23 (85.2)	61 (96.8)	0.125
Disease duration, month (median [IQR])	83.5 (42–132)	66 (21–120)	84 (59–132)	0.25
ESR, mm/hr (median[IQR])*	28.5 (17–41)	35 (23–56)	22 (14–38)	0.011
WBC, cells/mm^3^ (mean ± SD)	6,714 ± 2,600	7,031 ± 2,579	6,577 ± 2,618	0.818
ALC, cells/mm^3^ (mean ± SD)*	1665 ± 736	1369 ± 732	1791 ± 705	0.014
Hemoglobin, g/dL (mean ± SD)	12.0 ± 1.8	11.4 ± 2.4	12.3 ± 1.5	0.09
Platelet, cells/mm^3^ (mean ± SD)	232,000 ± 77,410	229,111 ± 97,242	233,523 ± 68,048	0.831
Serum creatinine, mg/dl (median[IQR])	0.71 (0.63–0.88)	0.68 (0.63–1.17)	0.72 (0.63–0.86)	0.250
UPCR (median[IQR])***	0.19 (0.11–0.39)	0.72 (0.25–2.44)	0.15 (0.1–0.23)	<0.001
Hypertension, n (%)	20 (22.2)	4 (14.81)	16 (25.34)	0.268
Clinical SLEDAI (median[IQR])***	0 (0–2)	4 (4–8)	0 (0–0)	<0.001
SLEDAI-2K (median[IQR])***	2 (0–4)	6 (4–10)	0 (0–2)	<0.001
Treatment				
Prednisolone usage, n (%)***	60 (66.7)	27 (100)	33 (52.4)	<0.001
Hydroxychloroquine usage, n (%)	74 (82.2)	20 (74.1)	54 (85.7)	0.231
Azathioprine usage, n (%)	31 (34.4)	6 (22.2)	25 (39.7)	0.11
Cyclophosphamide usage, n (%)	8 (8.9)	5 (18.5)	3 (4.8)	0.05
Mycophenolate mofetil usage, n (%)*	18 (20)	10 (37)	8 (12.7)	0.01
Tacrolimus usage, n (%)*	3 (3.3)	3 (11.1)	0 (0)	0.025

ESR, erythrocyte sedimentation rate; ALC, absolute lymphocyte count; UPCR, urine protein to creatinine ratio; SLEDAI, Systemic lupus erythematosus disease activity index; *p < 0.05, **p < 0.01, ***p < 0.001.

### Serum biomarkers in the different stages of SLE disease activity

The serum level of C3 had a tendency to be reduced, and anti-dsDNA was slightly higher in the active clinical SLE. However, the levels of these biomarkers in both active and inactive clinical SLE did not show a statistically significant difference (Table 2). In contrast, the active clinical SLE patients showed significantly elevated levels of IL-6 and CIC compared with the inactive ones (Table 2). The percentage of ds-DNA antibodies and complements in the SLE cohort is shown (Supplementary Table S2).

**Table 2:** Serum biomarkers in the different stages of SLE disease activity.

Biomarkers	Clinical Active (n = 27)	Clinical Inactive (n = 63)	*p-value*
C3, µg/ml	877 (694–1070)	1010 (824–1230)	0.107
C4, µg/ml	192 (120–246)	217 (144–282)	0.25
Anti-dsDNA, IU/ml	101.4 (37.2–436.5)	48.9 (10.0–209.8)	0.25
IL-6, pg/ml*	5.5 (4.0–14.5)	3.6 (2.6–6.7)	0.011
CIC, RU/ml**	10.12 (3.0–23.6)	2.1 (2.0–7.7)	0.001

Data showed in median (IQR). *p < 0.05, **p < 0.01.

### Correlation between serum biomarkers with SLE disease activity

The correlation of biomarkers with the modified SLEDAI-2K and the SLEDAI-2K is shown (Table 3). The erythrocyte sedimentation rate (ESR), IL-6, CIC, and anti-dsDNA showed a significant positive correlation with the modified SLEDAI-2K (R = 0.551, p < 0.001; R = 0.313, p = 0.003; R = 0.331, p = 0.001; and R = 0.211, p = 0.046, respectively), while both C3 and C4 did not show significant correlation **(**Table 3**)**. On the other hand, IL-6 did not reveal the correlation with the SLEDAI-2K while the other biomarkers (CIC, anti-dsDNA, C3, C4, and ESR) correlated with the SLEDAI-2K (R = 0.473, p < 0.001; R = 0.602, p < 0.001; R = −0.577, p < 0.001; R = −0.494, p < 0.001, and R = 0.468, p < 0.001, respectively) (Table 3).

**Table 3:** Correlation between serum biomarkers and SLEDAI score.

Serum biomarkers	Modified SLEDAI-2K		SLEDAI-2K	
	Correlation coefficient	*p-value*	Correlation coefficient	*p-value*
C3	−0.203	0.055	−0.577	<0.001***
C4	−0.180	0.089	−0.494	<0.001***
Anti-dsDNA	0.211	0.046*	0.602	<0.001***
ESR	0.551	<0.001***	0.468	<0.001***
IL-6	0.313	0.003**	0.153	0.149
CIC	0.331	0.001**	0.473	<0.001***

*p < 0.05, **p < 0.01, ***p < 0.001.

### Correlation of Individual Biomarkers

Levels of IL-6 did not display significant correlation with other biomarkers (CIC, C3, C4, and anti-dsDNA) except ESR (R = 0.444, p < 0.001) (Table 4). However, CIC significantly correlated with C3 (R = −0.396, p < 0.001), C4 (R = −0.457, p < 0.001), and anti-dsDNA (R = 0.484, p < 0.001) (Table 4).

**Table 4:** Correlation of individual biomarkers

Biomarkers	Statistical Analysis	C3	C4	Anti-dsDNA	ESR	CIC
IL-6	Correlation-coefficient	0.066	0.061	0.101	0.444	0.117
	p value	0.531	0.567	0.345	< 0.001***	0.272
CIC	Correlation-coefficient	−0.396	−0.457	0.484	0.256	
	p value	<0.001***	<0.001***	<0.001***	0.015*	

*p < 0.05, **p < 0.01, ***p < 0.001.

### Distinction of active clinical SLE using various biomarkers

**Figure 1:**
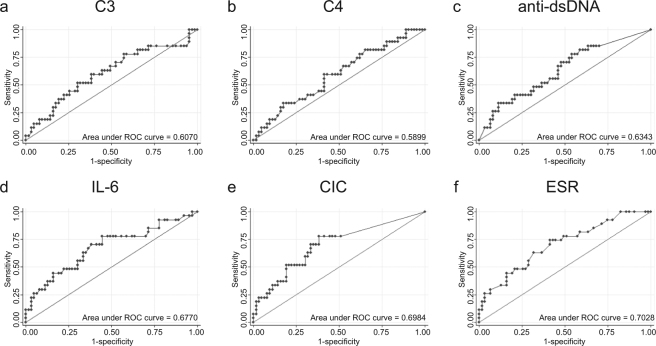
Receiver operating characteristic (ROC) curve and area under ROC curve of the following biomarkers; (**a**) Complement 3 (C3); (**b**) Complement 4 (C4); (**c**) anti-dsDNA; (**d**) Interleukin-6 (IL-6); (**e**) Circulating immune complex (CIC); (**f**) Erythrocyte sedimentation rate (ESR).

The area under the curve (AUC) of the ROC curve was analyzed to assess the performance of individual serum biomarkers to differentiate active clinical SLE. Based on the AUC of these biomarkers, CIC and IL-6 showed better parameters for determining active SLE than anti-dsDNA, C4 and C3 (AUC = 0.698, 0.677, 0.634, 0.410 and 0.393 respectively). Remarkably, ESR exhibited the highest AUC (AUC = 0.7028) in discriminating active SLE (Fig. 1). The optimal cut-off point for CIC is 4 RU/ml which gives a 70% sensitivity and 67% specificity respectively, whereas the cut-off for IL-6 is 4.35 pg/ml, which gives a sensitivity of 70% and specificity of 62% respectively.

### Biomarker models predict active clinical SLE

Among each biomarker tested, CIC yielded the highest sensitivity, specificity and odds ratio in predicting active clinical SLE (Table 5). The sensitivity of ESR is quite high but the specificity and OR in predicting disease flare is insignificant (Table 5**)**. Using a combination of different biomarkers increased the sensitivity to identify active clinical SLE and reduced specificity as well (Table 5). However, the combination of CIC or IL-6 generated a sensitivity much higher than the routine use of either low complement or anti-dsDNA (92.6% vs. 74.1%), while the specificity was comparable (47.6% vs. 52.4%) (Table 5). Also, the combination of IL-6 or CIC demonstrated the highest odds ratio of predicting active clinical SLE compared to the combination of complement or anti-dsDNA (OR = 7.27, 95%CI (1.99–26.63), p = 0.003 vs. OR = 3.14, 95%CI (1.16–8.48), p = 0.024) (Table 5). Although ESR showed a moderate degree of correlation with IL-6 (Table 4), the combination of ESR and CIC did not show a significant OR in predicting active disease (OR = 3.45, 95%CI (0.93–12.87), p = 0.065) (Table 5**)**.

**Table 5:** Sensitivity and specificity of biomarker models in the active clinical SLEDAI.

Biomarker models	Sensitivity	Specificity	OR	95%CI	*p-value*
Low complement (C3 < 900 µg/ml or C4 < 90 µg/ml)	51.8	65.1	2.01	(0.80–5.01)	0.136
anti-dsDNA (>100 IU/ml)	51.8	63.5	1.87	(0.75–4.67)	0.178
Low complement or anti-dsDNA*	74.1	52.4	3.14	(1.16–8.48)	0.024
IL-6 (>4.35 pg/ml)**	70.4	61.9	3.86	(1.46–10.18)	0.006
CIC (>4 RU/ml)**	70.4	66.7	4	(1.54–10.41)	0.004
IL-6 or CIC**	92.6	47.6	7.27	(1.99–26.63)	0.003
Low complement, anti-dsDNA, IL-6, or CIC*	96.3	28.6	5	(1.07–23.33)	0.041
ESR (>20 mm/hr)	77.78	42.86	2.63	(0.93–7.39)	0.068
ESR or CIC	88.89	30.16	3.45	(0.93–12.87)	0.065
Low complement, anti-dsDNA, ESR	88.89	19.05	1.88	(0.49–7.30)	0.36
Low complement, anti-dsDNA, ESR, CIC	88.89	15.87	1.51	(0.38–5.98)	0.558

*p < 0.05, **p < 0.01.

Also, seven out of twenty-seven of active clinical SLE cases showed normal levels of complement and anti-dsDNA. Up to 85% (6/7) had either elevated CIC or IL-6. The clinical manifestations included rash, lymphopenia, autoimmune hemolytic anemia (AIHA), psychosis, myelitis and mild lupus nephritis (Table 6).

**Table 6:** Clinical features and SLEDAI-2K of clinically active SLE patients with normal complement and anti-dsDNA.

Clinical symptoms	ID-1	ID-2	ID-3	ID-4	ID-5	ID-6	ID-7
Seizure	−	−	−	−	−	−	−
Psychosis	−	−	−	−	−	**+**	−
Organic brain syndrome	−	−	−	−	−	−	−
Visual disturbance	−	−	−	−	−	−	−
Cranial nerve disorder	−	−	−	−	−	−	−
Lupus headache	−	−	−	−	−	−	−
CVA	−	−	−	−	−	−	−
Vasculitis	−	−	−	−	−	−	−
Arthritis	−	−	−	−	−	−	−
Myositis	−	−	−	−	−	−	−
Urinary casts	−	−	−	−	−	−	−
Hematuria	−	−	−	**+**	−	−	−
Proteinuria	−	**+**	**+**	−	−	−	**+**
Pyuria	−	−	−	−	−	−	−
Rash	**+**	−	−	−	−	−	−
Alopecia	−	−	**+**	−	**+**	−	−
Mucosal ulcers	−	−	−	−	−	−	−
Pleurisy	−	−	−	−	−	−	−
Pericarditis	−	−	−	−	−	−	−
Low complement	−	−	−	−	−	−	−
Increased DNA binding	−	−	−	−	−	−−	−
Fever	−	−	−	−	−	−	−
Thrombocytopenia	−	**+**	−	−	−	−	−
Leukopenia	−	−	−	−	−	−	−
**SLEDAI-2K score**	**2**	**5**	**6**	**4**	**2**	**8**	**4**
Increased IL-6	+	+	+	−	−	+	−
Increased CIC	+	+	+	+	+	−	−
Other clinical SLE	−	AIHA, Lymphopenia	AIHA, Transverse myelitis, Lymphopenia	Lymphopenia	−	Lymphopenia	−

## Discussion

The current biomarkers (C3, C4, and anti-dsDNA) routinely used in clinical practice do not always correlate with the clinical manifestations of SLE patients. Complement can be changed by various factors and may not represent active clinical SLE^20^. The discovery of biomarkers for lupus nephritis has been extensively studied in order to identify better markers to predict disease flare^21–23^. The data of biomarkers for active non-renal SLE is quite limited, with minimal validation. Thirty percent of the patients in this cross-sectional study were clinically active, and forty-four percent of this active group were active in renal manifestation. The active SLE patients showed a significant difference of urine protein creatinine ratio compared to inactive group (p < 0.001) but complement and anti-dsDNA did not differ between the two groups. Surprisingly, there was no significant correlation between C3 and C4 levels with clinical SLEDAI in this study.

IL-6 plays a critical role in the B cell hyperactivity and immunopathology of human SLE and may have a direct role in mediating tissue damage^24^. However, human SLE data show various results. Serum levels of IL-6 are elevated in human SLE and have correlated with disease activity^9–11,25^, while another study reports the increase of serum levels of IL-6 in SLE with active hematological disease, but did not correlate with other organ involvement evaluated by the BILAG index^14^. Here we demonstrated the serum levels of CIC and IL-6 in the active SLE patients were significantly higher than those in the inactive SLE patients. Interestingly, we found the levels of IL-6 showed significant statistical correlation with clinical SLEDAI or modified SLEDAI-2K, but not with SLEDAI-2K, whereas CIC and anti-dsDNA correlated with both clinical SLEDAI or modified SLEDAI-2K. The reasons for this divergence could derive from the fact that IL-6 did not correlate with complement and anti-dsDNA criteria that are included in SLEDAI-2K.

The presence of DNA-anti-DNA immune complexes correlates with SLE disease activity, and deposits of these complexes have been demonstrated in affected tissues^26^. Patients with lupus nephritis were reported to have a higher level of circulating soluble immune complexes than the ones without renal involvement; however, the study was conducted with only twenty-one patients^27^. Serum levels of CIC, which contain a fragment of complement C1q, were tested in these SLE patients and showed correlation with clinical SLEDAI and SLEDAI-2K which represented overall SLE disease activity (not just renal manifestation).

ESR is associated with disease activity in SLE measured by the SELENA-SLEDAI^28^. A change in ESR between two visits was correlated with a change in disease activities; however the change did not reach statistical significance^28^. Such a result is similar to our findings that ESR was significantly higher in active SLE and was correlated with disease activity, but the OR for predicting disease flare was insignificant.

ROC curve analysis suggested that serum levels of ESR, CIC and IL-6 were satisfactory discriminators for determining active or inactive SLE (based on clinical SLEDAI score). Of interest, our study showed that ESR presented the highest AUC in discriminating active SLE. These data suggested that ESR, CIC and IL-6 may be used as alternative biomarkers for determining SLE activity. A correlation between ESR and IL-6 has been reported^29^. It will be more practical in the clinical setting if ESR can be used as an alternative biomarker instead of IL-6. However, the analysis showed that IL-6 gave a significant OR for predicting disease activity while ESR did not.

Pathogenesis of lupus disease is complex, and many mechanisms can contribute to the same phenotypes. The fact that CIC did not correlate with IL-6 but both markers did correlate with clinical activity suggested that a single biomarker may not be the ideal way to predict SLE disease activity. Next, we tried to analyze the biomarker models that could be the best predictor for disease flare. We selected the optimal cut-off point from the ROC curve of CIC as 4 RU/ml, which gives a sensitivity of 70% and specificity of 67% respectively, whereas the IL-6 cut-off is 4.35 pg/ml, which gives a sensitivity of 70% and specificity of 62% respectively. The cut-off points of ESR **(**>20 mm/hr), anti-dsDNA (>100 IU), C3 (<900 µg/ml), and C4 (<90 µg/ml) were the levels used in routine practice. If the threshold was adjusted, the OR may increase and provide better prediction for disease flare. We compared the sensitivity, specificity and odd ratios of combinations of individual markers. Models of either IL-6 or CIC showed the highest odd ratios for predict disease flare.

According to the SLE pathogenesis which involves both immune complex-mediated tissue injury and production of inflammatory cytokines (which may not only be limited to IL-6), the biomarkers that represent both processes should be used together to augment sensitivity in identifying disease flare. This idea was confirmed with the result that the combination of all serum biomarkers granted the highest sensitivity in detecting active clinical disease. The more markers used in the model, the higher was the sensitivity seen; however, the specificity was reduced.

Up to 70% of the patients in this study were clinically inactive, and some of them showed a rise of CIC and IL-6. We do not know whether those elevated levels happened before a disease flare or occurred because of a non-specific SLE condition such as non-obvious infection. A longitudinal study of SLE patients and controls from the non-autoimmune condition will provide more evidence to precisely determine the specificity of CIC and IL-6 in the prediction of SLE flare. Also, the twenty-five percent of patients (7/27) with active clinical SLE showed a normal level of complement and anti-dsDNA. These patients showed clinical active non-renal manifestations (such as rash, lymphopenia, AIHA, psychosis, and myelitis) and the elevation of either CIC or IL-6.

In conclusion, the heterogeneity of SLE pathogenesis is the crucial factor that impacts various clinical phenotypes which subsequently influence the types of biomarkers to use for monitoring disease activity and the selection of specific treatments that suit individual patients. A combination of biomarkers involved in immune-complex mediated processes (such as CIC) and cytokine-mediated inflammation (such as IL-6) can increase the chance to predict SLE disease activity. We propose that a biomarker model of CIC and IL-6, based on the highest odds ratio, may be suitable for routine use as additional markers to monitor SLE disease activity.

## Methods

### Study population and data collection

Ninety SLE patients who followed up at Ramathibodi Hospital, Bangkok, Thailand during 2014–2015 were enrolled. All of the patients were older than 18 years and met 1997 ACR criteria^30^ or SLICC criteria 2012^31^ for the classification of SLE. The collected data were demographic information, disease manifestations, laboratory tests and history of treatment. The exclusion criteria were SLE patients with overlapping syndromes, active infection, cancer or previous history of cancer, and allergic disease. The study complied with the Declaration of Helsinki which was used by the Faculty of Medicine at Ramathibodi ethics committee in approving the research protocol. All patients (or their legally authorized representative) gave written informed consent.

### Measurement of SLE disease activity

The Systemic Lupus Erythematosus Disease Activity Index **(**SLEDAI**)** is a global disease activity index that is widely used to monitor SLE disease activity^32^. Several versions of SLEDAI have been modified to make it more practical to use on a daily basis in clinical practice. Modification of SLEDAI (SLEDAI-2K) highly correlates with SLEDAI and can be used in clinical studies in SLE^32^. The modified SLEDAI-2K shows a good correlation with SLEDAI-2K^33^. Physicians evaluated the SLEDAI-2K in each SLE patient visiting the lupus clinic. The modified SLEDAI-2K, which did not include the low complement and the rise of anti-dsDNA, were analyzed as well. Active disease was defined if the Clinical SLEDAI score or modified SLEDAI-2K ≥ 1, and inactive disease if the Clinical SLEDAI score or modified SLEDAI-2K = 0.

### Measurement of anti-dsDNA, interleukin-6 (IL-6) and circulating immune complex (CIC) levels

Anti-dsDNA levels were measured using an enzyme-linked immunosorbent assay (ELISA) method, following the manufacturer’s recommended protocol (Euroimmun, Luebeck, Germany). Levels of IL-6 in the serum of SLE patients were measured using an electrochemiluminescence immunoassay (ECLIA) method (Roche Diagnostics GmbH, Mannheim, Germany). Circulating immune complex (CIC) levels were measured using an enzyme-linked immunosorbent assay (ELISA) method which provides a quantitative *in vitro* assay for human C1q-binding circulating immune complexes containing IgG antibodies (Euroimmun, Luebeck, Germany).

### Statistical analysis

Statistical analysis was performed using PASW Statistics 18 computer software. The mean ± SD was used to describe normally distributed data, median and IQR for skewed data, and proportion (%) was used to describe categorical data. Student’s *t-*test and Chi-square test/ Fisher’s exact test were used for evaluating continuous and categorical data, respectively. Spearman’s rank correlation was used to examine the correlation coefficient between parameters. Receiver operating characteristic (ROC) curves of ESR, IL-6, CIC, anti-dsDNA, C3, and C4 discriminated between active and inactive SLE. The cut-off of IL-6 and CIC that yielded the best sensitivity and specificity were determined from the ROC curve. The sensitivity and specificity of ESR, anti-dsDNA, C3, and C4 were analyzed using the standard cut-off levels routinely used in clinical practice. Logistic regression analyses were performed to predict clinically active SLE. The results were considered as statistically significant if the *p-*value was < 0.05.

### Data Availability

The datasets generated during and/or analysed during the current study are not publicly available due to the privacy protection for patients but are available from the corresponding author on reasonable request.

## Electronic supplementary material

Supplementary Information
