# Supplementary material for: The model of circulating immune complexes and interleukin-6 improves the prediction of disease activity in systemic lupus erythematosus
Source: Sci Rep. 2018 Feb 8;8:2620. doi: 10.1038/s41598-018-20947-4 (PMC5805742; doi:10.1038/s41598-018-20947-4)
Supplement: Supplementary file 1 — Supplementary Information [file 41598_2018_20947_MOESM1_ESM.pdf]

Supplementary Information

The model of circulating immune complexes and interleukin-6 improves the prediction of disease activity in systemic lupus erythematosus

CHOCKCHAI THANADETSUNTORN[1], PINTIP NGAMJANYAPORN[1], CHAVACHOL SETTHAUDOM[2], KENNETH HODGE[3], NISARA SAENGPIYA[2], and PRAPAPORN PISITKUN[1*].

[1]Division of Allergy Immunology and Rheumatology, Department of Medicine, Faculty of Medicine Ramathibodi Hospital, Mahidol University, Rama VI Road, Bangkok 10400, Thailand

[2]Immunology Laboratory, Department of Pathology, Faculty of Medicine Ramathibodi Hospital, Mahidol University, Rama VI Road, Bangkok 10400, Thailand

[3]Center of Excellence in Systems Biology, Faculty of Medicine, Chulalongkorn University, Bangkok 10330, Thailand

***Corresponding author**: Dr. Prapaporn Pisitkun, Department of Medicine, Faculty of Medicine Ramathibodi Hospital, 270 Rama VI Road, Ratchathewi, Bangkok, Thailand 10400

Email: prapaporn.pis@mahidol.ac.th

Supplementary Table 1. The prevalence of activity of all 24 descriptors of SLEDAI-2K for the studied cohort

SLEDAI-Score	Descriptor	Prevalence (%)
8	Seizure	0/90 (0)
8	Psychosis	1/90 (1.1)
8	Organic Brain Syndrome	0/90 (0)
8	Visual Disturbance	0/90 (0)
8	Cranial nerve Disorder	0/90 (0)
8	Lupus Headache	0/90 (0)
8	CVA	0/90 (0)
8	Vasculitis	3/90 (3.3)
4	Arthritis	2/90 (2.2)
4	Myositis	0/90 (0)
4	Urinary Casts	1/90 (1.1)
4	Hematuria	5/90 (5.5)
4	Proteinuria	16/90 (17.8)
4	Pyuria	1/90 (1.1)
2	New Rash	4 (4.4)
2	Alopecia	5 (5.5)
2	Mucosal Ulcers	2 (2.2)
2	Pleurisy	1/90 (1.1)
2	Pericarditis	0/90 (0)
2	Low Complement	36 (40)
2	Increased DNA binding	35 (38.9)
1	Fever	1/90 (1.1)
1	Thrombocytopenia	3/90 (3.3)
1	Leukopenia	1/90 (1.1)

Supplementary Table 2. The percentage of ds-DNA antibodies and complements in the SLE cohort

Tests		Clinical active (N=27) n/N, (Percentage)	Clinical inactive SLE (N=63) n/N, (Percentage)
Complement	Normal	14/27, (51.9)	41/63, (65.1)
	Low	13/27, (48.1)	22/63, (34.9)
Anti-dsDNA	Normal	13/27, (48.1)	42/63, (66.7)
	High	14/27, (51.9)	21/63, (33.3)
